# Taxonomic reconsideration of *Prunusveitchii* (Rosaceae)

**DOI:** 10.3897/phytokeys.115.29219

**Published:** 2019-01-17

**Authors:** Baohuan Wu, Chaoyu Liu, Daniel Potter, Dafang Cui

**Affiliations:** 1 College of Forestry and Landscape Architecture, South China Agricultural University, Guangzhou 510642, China South China Agricultural University Guangzhou China; 2 Department of Plant Sciences, University of California, One Shields Avenue, Davis, CA 95616, USA University of California Davis United States of America

**Keywords:** *
Prunus
*, *Prunusserrulata* var. *pubescens*, typification, synonyms, China

## Abstract

*Prunusveitchii* was published in 1912 and was treated as a synonym of P.serrulatavar.pubescens. The information about this taxon is relatively scarce. When consulting specimens of *Prunus* L., type materials of *Prunusveitchii* were found to belong to three taxa and *P.veitchii*, *P.concinna*, P.japonicavar.zhejiangensis, *C.jingningensis* and *C.xueluoensis* were found to be conspecific. The taxonomic status of *P.veitchii* is reconsidered in the present paper. Morphometric analyses were performed to evaluate the significance of differences between *P.veitchii* and P.serrulatavar.pubescens. The results show that the leaves of *P.veitchii* are significantly smaller and narrower than the leaves of P.serrulatavar.pubescens and the peduncle and pedicels are shorter. According to the results of morphometric analyses, *P.veitchii* should be treated as a separate species. To address these results, a lectotype of *P.veitchii* is designated here and *P.concinna, Cerasusjingningensis* and *C.xueluoensis* are here designated as synonyms of *P.veitchii*.

## Introduction

*Cerasus* A. Gray, the taxon that includes species commonly known as cherries, is a group that is famous for germplasm resources of edible fruits and flowering trees and shrubs. Historically, Cerasus has been treated either as a subgenus of Prunus L. or as a separate genus (Wen et al. 2008). In the past twenty years, molecular phylogenetic analyses (Bortiri et al. 2001; Lee and Wen 2001; Wen et al. 2008; Shi et al. 2013; Chin et al. 2014) have supported recognition of *Prunussensu lato*, including *Cerasus*, as a single genus and have also shown that, with the removal of the species in sect. Microcerasus, a monophyletic *Cerasus* can be recognised. Although the inclusion of *Cerasus* within *Prunus* is no longer as controversial as it used to be, there are still many problems with the taxonomy of this clade (Wu et al. 2018).

*Prunusveitchii* Koehne (Koehne 1912) is a species of shrub cherry that occurs at altitudes above 1000 m in western Hubei Province, China. It was treated as a synonym of P.serrulatavar.pubescens Wilson by Wilson (1916), a treatment followed by “Flora Reipublicae Popularis Sinicae” (Yü and Li 1986) and “Flora of China” (Li and Bartholomew 2003) and also by Koehne (1917), albeit with reservation. We found that the type materials of *P.veitchii* actually belonged to three taxa and that the voucher of Wilson’s treatment is not the same plant as the specimen on which Koehne’s description was based. This means that the taxonomic status of *P.veitchii* needs to be redefined.

Meanwhile, we also found that *P.veitchii*, *P.concinna*, *P.japonicavar.zhejiangensis*, *Cerasusjingningensis* and *C.xueluoensis* should all be conspecific due to their similarities in morphology and habitat. The histories of all of these taxa are relevant and are described below.

**Figure 1.**
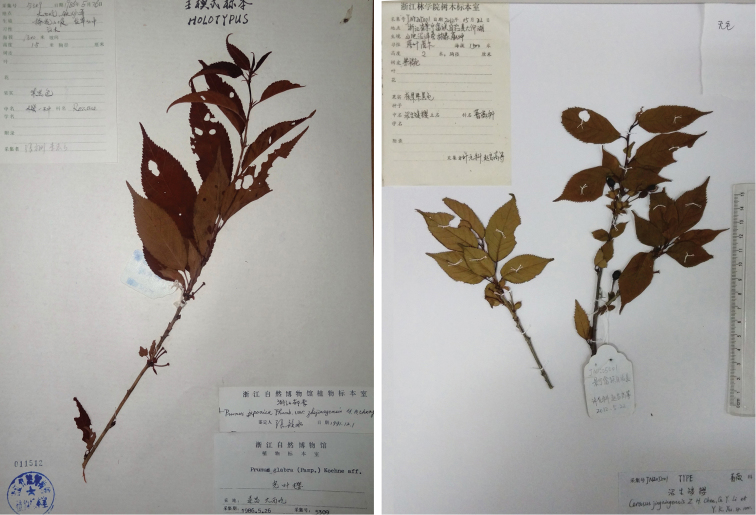
Holotypes of Prunusjaponicavar.zhejiangensis (left, photograph by Fanggang Zhang) and *Cerasusjingningensis* (right).

First, along with the publication of *P.veitchii*, Koehne (1912) described another shrub cherry, *P.concinna*, from a similar habitat. Due to the lack of materials, Koehne was uncertain about its status and the name is still unresolved today. Second, Chang (1992) described P.japonicavar.zhejiangensis based on Zhang Fanggang & Li Zhiyun 5309, which was collected from southern Zhejiang Province. This variety (Figure 1) was thought to be different from the typical variety in its persistent ovate stipules and black fruit (Chang 1992). However, it is strange that Chang did not include this variety in “Flora of Zhejiang” (Editorial Board 1993), which was published in the following year and for which Chang was involved in compiling most of the content for Rosaceae, including *Prunus* L. Although the taxon was later included in “Flora of China” (Li and Bartholomew 2003), it was overlooked in later publications (Wang 2014, Yan et al. 2017). Third, Xu et al. (2012) described a new species of cherry, *C.jingningensis* (Fig. 1), based on specimens collected from southern Zhejiang Province. Recently, P.japonicavar.zhejiangensis was treated as a synonym of *C.jingningensis* by Liu et al. (2017). Finally, *C.xueluoensis* was published by Nan et al. (2013) based on Cheng-Hui Nan 040301, which was collected from western Hubei Province.

Here, we use morphometric analyses to test the distinct nature of *P.veitchii* and P.serrulatavar.pubescens and conclude that the former should be recognised as a separate species. We designate a lectotype for *P.veitchii* and reduce *P.concinna*, *Cerasusjingningensis* and *C.xueluoensis* to its synonymy.

## Materials and methods

Herbarium specimens from A, AU, CSFI, DAV, E, GH, HBG, HHBG, HX, IBK, IBSC, IFP, JJF, JXU, K, KUN, LBG, MO, NAS, NF, NY, PE, UC, US, ZJFC and ZM (Chinese Academy of Sciences 2018, Thiers, [continuously updated].) were examined by visiting the herbaria or through the Chinese Virtual Herbarium (Chinese Academy of Sciences 2018) and Global Plant database (JSTOR 2018). To evaluate the differences between *P.veitchii* and P.serrulatavar.pubescens, specimens from different origins were selected to gather morphological data and which were subjected to morphometric analyses. Seven floral characters and eight leaf characters (Table 1) were selected for analyses, following Chang et al. (2007), though some characters used by Chang et al. (2007) were discarded because it was not possible to collect enough relevant data from the available specimens. A total of 26 specimens for floral characters and 44 specimens for vegetative characters were measured (see Appendix 1). Measurements were made manually with rulers for borrowed specimens or performed using Digimizer version 4.6.0 (MedCalc Software 2018) for online images.

**Table 1.** Floral characters and vegetative characters used in morphometric analyses.

**Code**	**Floral Character**	**Code**	**Vegetative Characters**
A	Peduncle length (cm)	H	Petiole length (cm)
B	Pedicel length (cm)	I	Leaf length (cm)
C	Length of calyx tube (cm)	J	Leaf width (cm)
D	Diameter of calyx tube top (cm)	K	Angle of leaf base (°)
E	Length of calyx lobe (cm)	L	Angle of leaf apex (°)
F	Width of calyx lobe (cm)	M	Length of leaf apex (cm)
G	Ratio of length and width of calyx lobe	N	Ratio of leaf length and petiole length
		O	Ratio of length and width of leaf

A non-parametric Kruskal-Wallis ANOVA was conducted to evaluate the significance of the difference in measured characters between *P.veitchii* and P.serrulatavar.pubescens in each character, as not all characters follow a normal distribution. Box plots were created to illustrate the differences. Data analyses were performed in R version 3.5.1 (R Core Team 2011) and diagrams were created by using ggplot2 package (Wickham 2016).

## Results

After examining the type specimens, other collections, relevant literature and plants in the field, we determined that *P.veitchii*, *P.concinna*, P.japonicavar.zhejiangensis, *C.jingningensis* and *C.xueluoensis* must be conspecific. Their original descriptions are not essentially different (Table 2). Although the type specimens of these taxa cannot all be compared directly because they were collected in different seasons and stages of development, it was clear that they are conspecific after consulting specimens collected from the type localities in different seasons.

**Table 2.** Characteristic description of *Prunusveitchii*, *P.concinna*, P.japonicavar.zhejiangensis, *Cerasusjingningensis* and *C.xueluoensis*, from the original literature (the description of *P.concinna* contains Koehne’s description (Koehne 1912) in the original literature and Rehder’s description (Rehder 1940) is based on the individuals introduced in Harvard Arnold Arboretum).

	* P. veitchii *	* P. concinna *	P. japonica var. zhejiangensis	* C. jingningensis *	* C. xueluoensis *
Life Form	Shrub	Shrub	Shrub	Shrub	Shrub, small tree
Lamina	–	narrow-elliptic to oblong-ovate, oblong-obovate	–	ovate, ovate-elliptic, obovate-elliptic	elliptic, obovate-elliptic
Leaf Length	–	3–6 (8) cm	–	3–6 cm	3–7 cm
Leaf Width	–	–	–	1.5–3 cm	1.5–3 cm
Leaf Margin	Incisively serrate	Sharply and rather finely serrate, doubly serrate	–	Acuminately serrate, biserrate	Serrate, biserrate
Leaf Apex	–	-	–	Acuminate, cuspidate	Acuminate, caudate
Leaf Base	–	Cuneate, rounded	–	Cuneate, rounded	Subrounded to broadly cuneate
Petiole	–	3–8 mm	–	4–10 mm	5–9 mm
Inflorescence	Umbellate, 1–3 flowered	Umbellate (Koehne, 1912), 1–4 flowered (Rehder, 1940), 1–2 flowered (Koehne, 1912)	–	Umbellate, subumbellate, 1–3 flowered	Umbellate, 2–4 flowered
Peduncle	No	No	–	Very short or no peduncle	Inconspicuous
Bract	Leaf like	–	–	Leaf like, subovate, ovate-oblong	Obovate, spatulate, fan-shaped, lobate
Pedicel	0.8–1.3 cm	0.8–1.5 cm (Rehder 1940), 0.8–0.9 cm (Koehne 1912)	–	0.8–1.8 cm	0.6–2.5 cm
Calyx Tube	Tubular with acute base, obconical, 8–10 mm long	Tubular (Rehder, 1940), obconically-tubular (Koehne, 1912), 9 mm long	–	Tubular-campanulate	Narrow tubular, apical enlarged, 6–10 mm long
Sepal	Ovate, oblong, entire	Ovate to ovate-oblong (Rehder 1940), ovate-triangular (Koehne 1912), entire	–	Ovate-triangular, entire	Ovate-triangular, entire
Published year	1912	1912	1992	2012	2013

**Figure 2.**
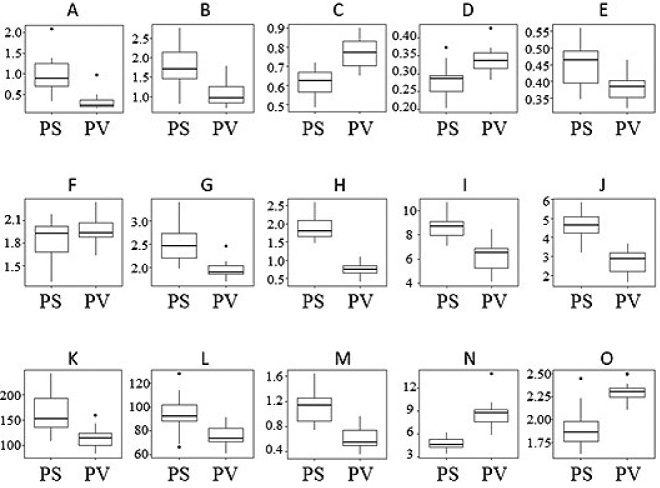
Univariate statistics with the minimum and maximum values for discriminating characters of *Prunusveitchii* and *P.serrulatavar.pubescens*. PS, P.serrulatavar.pubescens. PV, *P.veitchii*. A, Peduncle length (cm). B, Pedicel length (cm). C, Length of calyx tube (cm). D, Diameter of calyx tube top (cm). E, Length of calyx lobe (cm). F, Width of calyx lobe (cm). G, Ratio of length and width of calyx lobe. H, Petiole length (cm). I, Leaf length (cm). J, Leaf width (cm). K, Angle of leaf base (°). L, Angle of leaf apex (°). M, Length of leaf apex (cm). N, Ratio of leaf length and petiole length. O, Ratio of length and width of leaf.

The result of basic statistics and Kruskal-Wallis ANOVA are summarised in Table 3. The box plots (Figure 2) show that there is no significant overlap between *P.veitchii* and P.serrulatavar.pubescens for most of the measured characters. Moreover, ANOVA showed that the means of almost all measured characters differ significantly, except width of the calyx lobes.

**Table 3.** Arithmetic average ±standard deviation and Kruskal-Wallis ANOVA for measured morphological characters. A, Peduncle length (cm). B, Pedicel length (cm). C, Length of calyx tube (cm). D, Diameter of calyx tube top (cm). E, Length of calyx lobe (cm). F, Width of calyx lobe (cm). G, Ratio of length and width of calyx lobe. H, Petiole length (cm). I, Leaf length (cm). J, Leaf width (cm). K, Angle of leaf base (°). L, Angle of leaf apex (°). M, Length of leaf apex (cm). N, Ratio of leaf length and petiole length. O, Ratio of length and width of leaf.

Variates	* Prunus veitchii *	P. serrulata var. pubescens	Chi-Square value (ANOVA)	P value (ANOVA)
A	0.34±0.24	0.96± 0.44	12.639	<0.001
B	1.09±0.37	1.79± 0.45	9.536	<0.01
C	0.77±0.08	0.61± 0.07	14.158	<0.001
D	0.34±0.04	0.28± 0.04	7.424	<0.01
E	0.38±0.04	0.45± 0.06	6.869	<0.01
F	0.2±0.02	0.18± 0.03	0.925	0.364
G	1.95±0.22	2.5± 0.38	12.639	<0.001
H	0.74±0.16	1.88±0.31	30.6	<0.001
I	6.18±1.23	8.65±0.96	25.988	<0.001
J	2.71±0.59	4.62±0.67	29.021	<0.001
K	115.53±20.7	162.63±38.39	15.341	<0.001
L	75.9±8.61	94.39±13.18	19.991	<0.001
M	0.61±0.21	1.1±0.24	22.205	<0.001
N	8.55±1.89	4.68±0.71	30.069	<0.001
O	2.29±0.11	1.89±0.19	24.535	<0.001

## Discussion

*Prunusveitchii* was treated as a synonym of P.serrulatavar.pubescens by Wilson, a treatment that was followed by Koehne with reservation (Koehne 1917). Koehne (1917) mentioned that the sepals of *P.veitchii* are ovate and shorter and the leaflets are smaller than those of P.serrulatavar.pubescens. Consistent with Koehne’s observation, our morphometric analyses show that the leaves of *P.veitchii* are smaller, while the sepals are shorter and wider. The leaves of *P.veitchii* are also obviously narrower than the leaves of P.serrulatavar.pubscens, while the calyx tubes are longer and the peduncles and pedicels are shorter. These results indicate that *P.veitchii* should not be treated as a synonym of P.serrulatavar.pubescens.

The short peduncle was thought to be an important feature that distinguished *P.sargentii* Rehder from members of the *P.serrulata* complex (Chang et al. 2007). According to the key to classify the *P.serrulata* complex and its related species published by Chang et al. (2007), *P.veitchii* is similar to *P.sargentii*, having an umbellate or subumbellate inflorescence, sessile or short-pedunculate, consisting of 1–4 flowers with tubular hypanthia, triangular-lanceolate sepals with entire margins and white to reddish petals. Nonetheless, *P.veitchii* is definitely different from *P.sargentii*, which has small and elliptic or obovate-elliptic shaped leaves and short petioles, as opposed to the leaves of *P.sargentii* are elliptic-obovate or oblong-obovate and the length of leaves and petioles can reach 12 cm and 3 cm long (Rehder 1940). In addition, the distribution of *P.veitchii* is significantly different from that of *P.sargentii*. The former is mainly distributed around central and eastern China, while the latter is mainly distributed in northern Japan, the Korean peninsula and far eastern Russia (Chang et al. 2007). Therefore, we think that it is better treated as an separate species, based on current evidence.

E. H. Wilson 66 (Veitch Expedition) collected in April 1900, was cited as the voucher when Koehne described *P.veitchii*. However, this collection number is a source of some confusion. Number “66” was re-used by Wilson for a specimen collected in 1907 during his expedition for Arnold Arboretum, which was determined by Koehne (1912) as a certain form of *P.triflora*. Another number “66a”, also collected in April 1900, was cited as *P.tenuiflora* by Koehne in “Plantae Wilsonianae” (Koehne 1912). There are 7 sheets (Table 4) designated as Wilson 66, collected in April 1900, in the Global Plant database (JSTOR 2018), three of which are not congruent with the original description. Amongst these three specimens, one of them, A00241703, contains Wilson’s handwriting, which says ‘Prunusserrulatavar.pubescens’, indicating it is the voucher for Wilson’s treatment of *P.veitchii* as a synonym of P.serrulatavar.pubescens. It is reasonable to infer that the mixed collection led Wilson to propose a taxonomic treatment, different from Koehne.

**Table 4.** Type and original materials of *Prunusveitchii*, *P.concinna*, P.japonicavar.zhejiangensis, *Cerasusjingningensis* and *C.xueluoensis*.

Specimen	Type	Collecting locality	Identification
E. H. Wilson 66 – E00417568	Original material	W. Hubei	* P. veitchii *
E. H. Wilson 66 – HBG511147	Original material	W. Hubei	* P. veitchii *
E. H. Wilson 66 – Y00415930	Original material	W. Hubei	* P. veitchii *
E. H. Wilson 66 – US00130697	Original material (lectotype designated in this paper)	W. Hubei	* P. veitchii *
E. H. Wilson 66 – A00032230	–	W. Hubei	A small branch is *P.veitchii*, the other 3 branches are *P.pseudocerasus*
E. H. Wilson 66 – A00241703	–	W. Hubei	*P.tenuiflora* (P.serrulatavar.pubescence)
E. H. Wilson 66 – K000737109	–	W. Hubei	*P.tenuiflora* (P.serrulatavar.pubescence)
E. H. Wilson 2825	Type of *P.concinna*	W. Hubei	* P. veitchii *
Zhang Fanggang & Li Zhiyun 5309	Type of P.japonicavar.zhejiangensis	S. Zhejiang	* P. veitchii *
Y.K.Xu, C.G.Zhao etc. JN1205001	Type of *C.jingningensis*	S. Zhejiang	* P. veitchii *
Cheng-Hui Nan 040301	Type of *C.xueluoensis*	W. Hubei	* P. veitchii *

**Figure 3.**
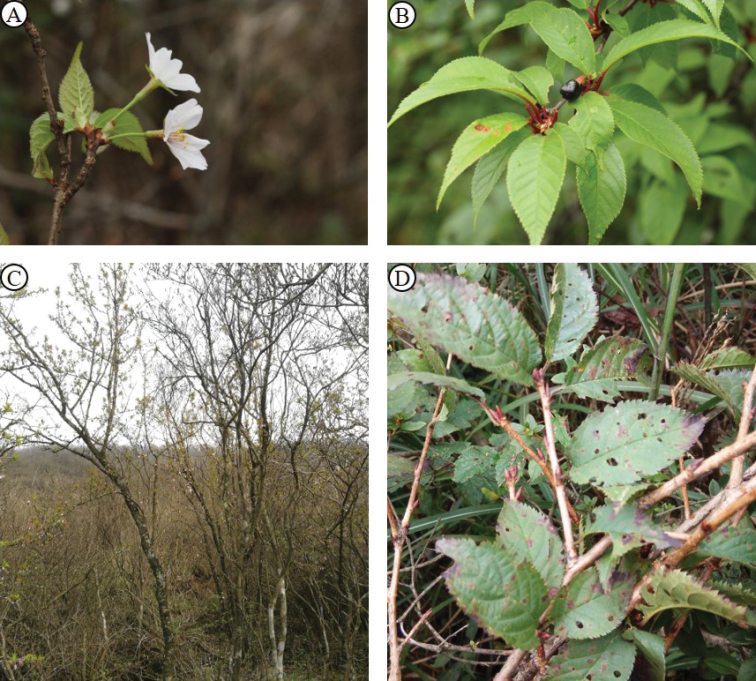
*P.veitchii*. A. Flower branch. B. Fruit Branch. C. Individual. D. Variation of the winter buds.

As for why this species was published again several times, we believe that there are several reasons besides the confusing voucher. First, the vouchers of this species are deposited in different herbaria in different countries, so it would have been difficult to consult all of them in the past. Second, few sources, especially those easily accessible to Chinese plant taxonomists, record this species. *P.veitchii* is not included in “Flora Hubeiensis” (Fu 2002) and it is listed as one of the synonyms of P.serrulatavar.pubescens in “Flora Reipublicae Popularis Sinicae” (Yü and Li 1986) and “Flora of China” (Li and Bartholomew 2003), which makes it easy to be ignored. And neither “Flora Hubeiensis” (Fu 2002) nor “Reipublicae Popularis Sinicae” (Yü and Li 1986) record *P.concinna*, which is only listed as a species that could not be treated in “Flora of China” (Li and Bartholomew 2003) because the authors had not seen the type specimens. Third, this species sometimes has three winter buds growing side by side, which has led some authors to treat it mistakenly as a member of section Microcerasus (Nan et al. 2013, Wang 2014, Liu et al. 2017). However, this trait is quite unstable. From observations of herbarium specimens and plants in the field, we found that the number of buds varies from one to three or four and mostly only one bud can be found (Figure 3).

According to the International Code of Nomenclature (ICN) (McNeill et al. 2012), it is necessary to designate a lectotype of *P.veitchii*, since the voucher points to more than one taxon. We choose the barcoded sheet US00130697 as the lectotype, since a label with Koehne’s handwriting, ‘*Prunusveitchii* Koehne’ is affixed to it.

## Taxonomic treatment

### Prunus veitchii

Taxon classificationPlantaeRosalesRosaceae

Koehne, Pl. Wilson. (Sargent) 1(2): 257. 1912

Figure 3

Type: China, western Hubei, April 1900, E.H. Wilson 66 (lectotype, designated here: US! [US00130697]; isolectotypes E! [E00417568], HBG! [HBG511147], NY! [NY00415930], A! [A00032230 in part]).
Prunus concinna Koehne, Pl. Wilson. (Sargent) 1(2): 210. 1912, syn. nov. Type: China, western Hubei, 7 April 1907, E.H. Wilson 2825 (holotype: K! [K000737137]).
Prunus japonica Thunb. var. zhejiangensis Y. B. Chang, Bull. Bot. Res. 12(3): 271–274, 1992. Type: China, Zhejiang, Suichang, Daixikeng, Tieluyang, 26 May 1986, F. G. Zhang & Z. Y. Li 5309 (holotype: ZM!).
Cerasus jingningensis Z. H. Chen, G.Y. Li & Y. K. Xu, Jour. of Zhejiang For. Sci. & Tech. 32(4): 81–83, 2012, syn. nov. Type: China, Zhejiang, Jingning She Autonomous County, Dayanghu, 22 May 2012, Y. K. Xu, C. G. Zhao et al. JN1205001 (holotype: ZJFC!).
Cerasus xueluoensis C. H. Nan & X. R. Wang, Ann. Bot. Fennici 50: 79–82, 2013, syn. nov. Type: China, Hubei, Enshi Tujia and Miao Autonomous Prefecture, Xuanen County, Xueluozhai, 3 April 2009 C. H. Nan 040301 (holotype: NF!).

#### Description.

Small trees, sometimes shrubs, deciduous, up to 3 m tall. Winter buds ovoid, apex acute, 1–3(4). Stipules lanceolate, sometimes ovate and lobed. Leaves elliptic to obovate-elliptic, 3–8 × 1.5–3.5 cm, apex acuminate, base subrounded to broadly cuneate, abaxially pale green and glabrous, sparsely pilose or sometimes pilose when young, adaxially green and glabrous or sparsely pubescent, margin serrate or biserrate. Petiole 4–10 mm, glabrous or sparsely pilose, apex with 2 nectaries or not. Inflorescence umbellate or sometimes corymbose, peduncle short or inconspicuous, 1–4-flowered, involucral bracts spatulate or obovate-elliptic, bracts ovate, obovate or spatulate, margin serrate. Pedicel 6–25 mm, glabrous or sparsely pilose. Hypanthium tubular, 6–10 × 1.5–3 mm, reddish-green to purplish, glabrous or sparsely pubescent. Sepals ovate-triangular to triangular-lanceolate, 3–5 mm, margin entire. Petals white or pinkish, obovate, apex emarginate, ca. 10 mm long. Stamens ca. 30–40. Style glabrous. Drupe ovoid or globose, ca. 8–10 mm in diam., glabrous, black when ripe. Flowering March-April, fruiting May-June.

#### Distribution and habitat.

**Figure 4.**
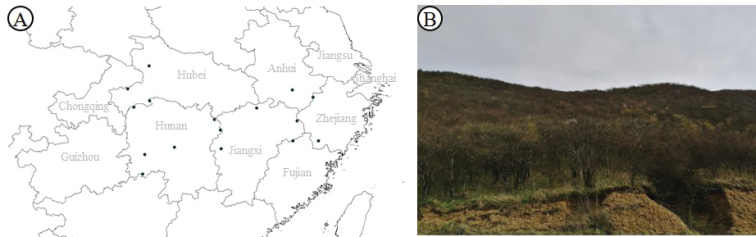
Distribution and habitat of *P.veitchii*. A. Distribution. B. Habitat.

Anhui, Fujian, Hubei, Hunan, Jiangxi, Zhejiang Provinces, usually occurs in mountain-top thickets at elevations of 800 to 1700 m (Figure 4).

#### Specimens examined.

**Fruit or leaf branch**, JianJun Zhou 16050702, Xunlin Yu & Hui Zhou 14051515 (CSFI); Fusong Peng 728, 551, Anonymous 23060, Anonymous & Qibai Xiang 844, Laiguan Lin 5976, Linhan Liu 1838, Xianyu He 21316, C. Y. Wu L72, Jiangxidiaochadui 348, Jiangxidui 1242 (PE); Anonymous 11758 (NAS); Choufen Liang 34522; 34484, 34442 (IBK); Xianyu He 23025, Wukaodui 2386 (IBSC); Yaoguo Xiong 07753, 08772 (LBG); Changming Xie et al. L8633-304, Jianshe Fang et al. L8635-320, Maochun Liu 840044, Chensen Ding & Xianglin Shen 5234, 5342, 5215 (ZJFC). **Flower branch**, H. H. Chung s. n. (AU); Xu Zhang 2015033003, Xunlin Yu, Fan Zhang, Ronghui Tu 16040517, Xunlin Yu, Si Feng, Fanxun Zhang 16040506 (CSFI); HZ017025 (HZ); Lai & Shan 647, Niemin Xiang 92022 (NAS); Anonymous 4218, Jiangxidui 81 (PE); Chensen Ding et al. 5008, Liang Chen 0219 (ZJFC).

## Supplementary Material

XML Treatment for Prunus veitchii

## Appendix

P. serrulata var. pubescens

Flower

China: Anonymous 118 (IFP), E. H. Wilson 20 (A00241699), E. H. Wilson 20 (A00241700), E. H. Wilson 13, 66, 69 (A), Anonymous s. n. (NAS00358158), H. F. Chow 40129 (PE), Z. Wang 2283 (PE).

North Korea: C. S. Chang & S. A. Ryue sky 0038 (PE).

Leaf

E. H. Wilson 20 (A00241699), E. H. Wilson 20 (A00241700), E. H. Wilson 13, 51, 51a, 69 (A), H. H. Chung s. n. (AU034505), Wilson 5833 (GH), Anonymous 3038 (NAS00358152), X. Y. He 21994 (NAS), M. B. Deng 4136 (NAS), T. Y. Zhou 1101 (NAS), K. Nakashima s. n. (NAS00358168), M. B. Deng 5498 (NAS), S. X. Li 592 (PE), T. Tang 1948 (PE).

P.serrulatavar.pubescens determined as *P.leveilleana*

Flower

Japan

S. Tsugaru 14295, 16109 (MO), S. Tsugaru et al. 32548 (MO), T. Sawada 895(MO), T. Sawada et al. 287 (MO), K. Seto 28312 (MO),

Leaf

Japan: C. Howick et al. HMT2688, HMT2689 (MO), S. Tsugaru 14326 (MO), S. Tsugaru et al. 18429 (MO), S. Tsugaru et al. 692 (MO), S. Tsugaru et al. 27511, 27680, 29096 (MO), S. Tsugaru & T. Takahashi 14586 (MO), T. Takahashi & G. Murata 2913 (MO), T. Sawada 895 (MO).

*
P. veitchii
*

Flower

China: E. H. Wilson 66 (HBG511147, NY00415930, US00130697), H. H. Chung s. n. (AU039954), Q. Z. Lin 054029 (CSFI), X. L. Yu et al. 16040517 (CSFI044820, CSFI044821, CSFI044822), S. Feng & G. X. Feng 16040517 (CSFI), X. Zhang 2015033004 (CSFI), Anonymous 547 (HHBG_ HZ017025).

Leaf

China: G. Yao & R. P. Jiang 11758 (NAS), Anonymous 660465 (LBG00010741), Y. G. Xiong 07753, 08772 (LBG), X. L. Yu & H. Zhou 14051515(CSFI), H. Zhou 16050702 (CSFI), C. F. Liang 34442, 34484, 34522 (IBK), Wukaodui 2386 (IBSC), X. G. Li 203583 (IBSC), X. Y. He 23025 (IBSC), F. S. Peng 728 (PE), Jiangxidui 348 (PE), Jiangxidiaochadui 348, 1242 (PE), Y. K. Xu, C. G. Zhao et al. JN1205001 (ZJFC).

## References

[B1] Bortiri E Oh SH Jiang J Baggett S Granger A Weeks C Buckingham M Potter D Parfitt DE (2001) Phylogeny and systematics of *Prunus* (Rosaceae) as determined by sequence analysis of ITS and the chloroplast trnL-trnF spacer DNA. Systematic Botany 26: 797–807.

[B2] Chang YB (1992) New plants from Zhejiang, China. Bulletin of Botanical Research 12: 271–274.

[B3] Chang KS Chang CS Park TY Roh MS (2007) Reconsideration of the *Prunusserrulata* complex (Rosaceae) and related taxa in eastern Asia. Botanical Journal of the Linnean Society 154(1): 35–54. 10.1111/j.1095-8339.2007.00631.x

[B4] Chin SW Shaw J Haberle R Wen J Potter D (2014) Diversification of almonds, peaches, plums and cherries – molecular systematics and biogeographic history of *Prunus* (Rosaceae). Molecular Phylogenetics and Evolution 76: 34–48. 10.1016/j.ympev.2014.02.024 24631854

[B5] Chinese Academy of Sciences (2018) Chinese Virtual Herbarium. http://www.cvh.org.cn/ [November 13, 2018]

[B6] Editorial Board (1993) Flora of Zhejiang, Vol. 3. Zhejiang Science and Technology, 239–261.

[B7] Fu SX (2002) Flora Hubeiensis. Vol. 2. Hubei Science & Technology Press, Wuhan, 217–233.

[B8] JSTOR (2018) Global Plants. https://plants.jstor.org/ [August 16, 2018]

[B9] Koehne BAE (1912) *Prunus*. In: Sargent CS (Ed.) PlantaeWilsonianae: an enumeration of the woody plants collected in western China for the Arnold arboretum of Harvard university during the years 1907, 1908, and 1910 by E H Wilson.The University press, Cambridge, 196–282.

[B10] Koehne BAE (1917) Die Kirschenarten Japans. In: Mitteilungen der Deutschen dendrologischen gesellschaft. Wendisch Wilmersdorf, 2–65.

[B11] Lee S Wen J (2001) A phylogenetic analysis of *Prunus* and the Amygdaloideae (Rosaceae) using ITS sequences of nuclear ribosomal DNA. American Journal of Botany 88(1): 150–160. 10.2307/2657135 11159135

[B12] Li CL Bartholomew B (2003) *Prunus*. In: Flora of China. Science Press & Missouri Botanical Garden Press, Beijing & St. Louis, 404–420.

[B13] Liu RL Zhang FG Chen WJ Chen F Chen ZH (2017) Noteworthy Plants in Prunoideae of Rosaceae from Zhejiang. Journal of Hangzhou Normal University 16: 518–521. [Natural Science Edition]

[B14] McNeill J Barrie FR Buck WR Demonlin V Greuter W Hawksworth DL Herendeen PS Knapp S Marhold K Prado J Prud’Homme Van Reine WF Smith GF Wiersema JH Turland NJ (2012) International Code of Nomenclature for Algae, Fungi, and Plants (Melbourne Code): adopted by the Eighteenth International Botanical Congress, Melbourne. Koeltz Scientific Books.

[B15] MedCalc Software (2018) Digimizer image analysis software. Ostend. http://www.digimizer.com

[B16] Nan CH Wang XR Tang GG Yi XG Luo SJ (2013) *Cerasusxueluoensis* (Rosaceae), a new species from China. Annales Botanici Fennici 50(1–2): 79–82. 10.5735/085.050.0114

[B17] R Core Team (2011) R: a language and environ-ment for statistical computing. The R Foundation for Sta-tistical Computing, Vienna. http://www.R-project.org/ [Accessed 10 November 2018]

[B18] Rehder A (1940) Manual of Cultivated Trees and Shrubs Hardy in North America Exclusive of the Subtropical and Warmer Temperate Regions. Second edition. MacMillan, New York, 452–481.

[B19] Shi S Li J Sun J Yu J Zhou S (2013) Phylogeny and classification of *Prunus**sensu lato* (Rosaceae). Journal of Integrative Plant Biology 55(11): 1069–1079. 10.1111/jipb.12095 23945216

[B20] Thiers B [continuously updated]. Index Herbariorum: A global directory of public herbaria and associated staff. New York Botanical Garden’s Virtual Herbarium. http://sweetgum.nybg.org/science/ih/

[B21] Wang XR (2014) An Illustrated Monograph of Cherry Cultivars in China. Science Press, Beijing, 130–132.

[B22] Wen J Berggren TS Lee C-H Ickert-Bond S Yi T Yoo K-O Xie L Shaw J Potter D (2008) Phylogenetic inferences in *Prunus* (Rosaceae) using chloroplast ndhF and ribosomal ITS sequences. Journal of Systematics and Evolution 46: 322–332.

[B23] Wickham H (2016) ggplot2: Elegant Graphics for Data Analysis. Springer-Verlag New York.

[B24] Wilson EH (1916) The cherries of Japan. University Press, Cambridge, 1–34.

[B25] Wu BH Huang WX Shi WT Yang HJ Cui DF (2018) Numerical taxonomy study on PrunusL.subgenusCerasus (Mill.) A. Gray in China. Acta Scientiarum Naturalium Universitatis Sunyatseni 57: 36–43.

[B26] Xu YK Zhao CG Yan BX Ma DD Chen ZH (2012) A new species of *Cerasus* Mill. from Zhejiang Province. Zhejiang Linye Keji 32: 81–83.

[B27] Yan CF Xu L Zhao Q Wang XT Sha CL (2017) Classification research of chinese native *Cerasus* resources. Journal of Jiangsu Forestry Science & Technology 44: 35–40.

[B28] Yü TT Li CL (1986) *Cerasus*. Flora Reipublicae Popularis Sinicae. Science Press, Beijing, 41–89.

[B29] Wang XR (2014) An Illustrated Monograph of Cherry Cultivars in China. Science Press, Beijing, 130–132.

